# DPI-ELISA: a fast and versatile method to specify the binding of plant transcription factors to DNA *in vitro*

**DOI:** 10.1186/1746-4811-6-25

**Published:** 2010-11-25

**Authors:** Luise H Brand, Tobias Kirchler, Sabine Hummel, Christina Chaban, Dierk Wanke

**Affiliations:** 1 ZMBP Pflanzenphysiologie, Universität Tübingen, Tübingen, Germany

## Abstract

**Background:**

About 10% of all genes in eukaryote genomes are predicted to encode transcription factors. The specific binding of transcription factors to short DNA-motifs influences the expression of neighbouring genes. However, little is known about the DNA-protein interaction itself. To date there are only a few suitable methods to characterise DNA-protein-interactions, among which the EMSA is the method most frequently used in laboratories. Besides EMSA, several protocols describe the effective use of an ELISA-based transcription factor binding assay e.g. for the analysis of human NFκB binding to specific DNA sequences.

**Results:**

We provide a unified protocol for this type of ELISA analysis, termed DNA-Protein-Interaction (DPI)-ELISA. Qualitative analyses with His-epitope tagged plant transcription factors expressed in *E. coli *revealed that EMSA and DPI-ELISA result in comparable and reproducible data. The binding of *At*bZIP63 to the C-box and *At*WRKY11 to the W2-box could be reproduced and validated by both methods. We next examined the physical binding of the C-terminal DNA-binding domains of *At*WRKY33, *At*WRKY50 and *At*WRKY75 to the W2-box. Although the DNA-binding domain is highly conserved among the WRKY proteins tested, the use of the DPI-ELISA discloses differences in W2-box binding properties between these proteins. In addition to these well-studied transcription factor families, we applied our protocol to *At*BPC2, a member of the so far uncharacterised plant specific Basic Pentacysteine transcription factor family. We could demonstrate binding to GA/TC-dinucleotide repeat motifs by our DPI-ELISA protocol. Different buffers and reaction conditions were examined.

**Conclusions:**

We successfully applied our DPI-ELISA protocol to investigate the DNA-binding specificities of three different classes of transcription factors from *Arabidopsis thaliana*. However, the analysis of the binding affinity of any DNA-binding protein to any given DNA sequence can be performed *via *this method. The DPI-ELISA is cost efficient, less time-consuming than other methods and provides a qualitative and quantitative readout. The presented DPI-ELISA protocol is accompanied by advice on trouble-shooting, which will enable scientists to rapidly establish this versatile and easy to use method in their laboratories.

## Background

The developmental processes and specific cellular responses of organisms are governed by differential gene expression. The transcriptional regulation of genes is directly dependent on the presence or absence of transcription factors, which are able to bind to specific short promoter elements. It is of major interest to unravel the interaction between eukaryote transcription factors and their DNA-motif companions. This question needs to be addressed from both, the protein and the DNA perspective. Several standard methods are routinely applied to identify DNA-binding proteins for an already known DNA sequence consensus [1,2]. One frequently used method is the yeast-one-hybrid screening approach: Hybrid-gene products bind specifically to the DNA sequence under investigation and thereby mediate reporter activation [2]. Subsequently, a detailed analysis of the binding specificity is essentially needed for proper characterisation of the transcription factor. Here, the most successful laboratory approach has been the *in vitro *electrophoretic mobility shift assay (EMSA) [3]. If the tested protein binds to radioactively labelled DNA-probes, a shifted band signal corresponding to the molecular weight of the DNA-protein complex is detected [3,4]. Because EMSA is based on gel electrophoresis technology, the amount and length of the DNA-probes to be analysed is limited. Although some other methods exist that assist in the elucidation of DNA-protein interactions, a routine method to address this question from the protein perspective is so far lacking [3,5]. The solution to this problem could be the DNA-protein-interaction enzyme-linked immunosorbent assay (DPI-ELISA). The basic design of the method was established for the first time about 15 years ago [6]. Since then, similar ELISA-based transcription factor assays to study DNA-protein interactions have been published [7-11]. While all kinds of transcription factors can potentially be studied by the DPI-ELISA, it has mainly been applied to investigate inflammatory responses triggered by NFκB in human [7,9,10]. Surprisingly, DPI-ELISA was not substituted for traditional EMSA in other research fields, although it was clear that the DPI-ELISA has some advantages over the traditional EMSA [6-10]: it does not rely on radioactive detection, displays a 10-fold increased sensitivity and provides a qualitative and quantitative readout. For example, there are only two publications in plant sciences that utilise DPI-ELISA to study bZIP proteins according to a protocol by Renard *et al*. [9,12,13].

Here, we evaluated different ELISA-based transcription factor assays from human research (Additional file 1) [6-10] to provide a unified laboratory scale DPI-ELISA protocol for plant transcription factors. For the first time we show that recombinant plant transcription factors yield comparable results with EMSA and with our DPI-ELISA protocol. Initially, we applied our protocol to verify results already published on *At*bZIP63 and *At*WRKY11 binding to the C-box and the W2-box, respectively. We demonstrate the versatile use of the DPI-ELISA by analysing the binding specificities of so far uncharacterised plant transcription factors (*At*WRKY33, *At*WRKY50 and *At*WRKY75, *At*BPC2). Finally, we provide a step-by-step overview on notes and advice on trouble-shooting.

## Materials and methods

### Plasmid construction

The cDNA of the desired transcription factor was amplified by PCR without a stop codon for subsequent cloning into pENTR/D-TOPO (Invitrogen). After sequencing, the specific insert was recombined *via *Gateway (Invitrogen) LR-reaction into appropriate destination vectors pET-DEST42 (Invitrogen) (*At*bZIP63, *At*WRKYs) or pET32b-GW (vector database MPI, Cologne, [14]) (*At*BPC2). As negative controls, untransformed BL21/RIL cells and cells transformed with empty vectors, that lack the Gateway cassette (Invitrogen), were included. The expressed fusion proteins used in this study were as follows: *At*bZIP63 - amino acids 1-314 of AT5G28770, C-terminal V5:His; *At*WRKY11^DBD ^- amino acids 229-325 of AT4G31550, C-terminal V5:His; *At*WRKY33^cDBD ^- amino acids 346-449 of AT2G38470, C-terminal V5:His; *At*WRKY50^DBD ^- amino acids 99-173 of AT5G26170, C-terminal V5:His; *At*WRKY75^DBD ^- amino acids 58-145 of AT5G13080, C-terminal V5:His; *At*BPC2 - amino acids 1-279 of AT1G14685, N-terminal His.

### Protein expression and protein extraction for DPI-ELISA

Different expression systems and protein extraction methods are compatible with the described DPI-ELISA. We used the *E. coli *strain BL21/RIL for protein expression. The cells were grown over night in selective liquid media and diluted 1:20 in LB-medium without antibiotics the next day. At 2 hours after dilution protein expression was induced by the addition of 1 mM of IPTG. The cells were collected by centrifugation (2500 g, 20 min, 4°C) at an optical density E_600 _= 1 (4-6 hours) and washed (10 mM Tris-HCl pH7.5-8, 100 mM NaCl). The cell sediment was resuspended in protein extraction buffer (4 mM HEPES pH 7.5, 100 mM KCl, 8% (v/v) glycerol, 0.2% biotin-free BSA, 5 mM dithiothreithol (DTT), 1× complete proteinase inhibitor without EDTA (Roche)). NOTE: *DTT and BSA need to be added after measuring the total protein amount of the extracts via Bradford Assay*. Native protein extraction was performed by sonication (*At*WRKYs, *At*BPC2) [6,8]. The total protein amount of the extracts was measured *via *Bradford Assay (BioRad). The protein extracts were kept either at 4°C ready to use or at -20°C. If stored at -80°C, glycerol is added to a final concentration of 20% (v/v). The presence of proteins was verified by SDS-PAGE and western blotting [15].

### Electrophoretic mobility Shift Assay

EMSAs were performed essentially according to previous publications [13,14,16]. The recombinant proteins (*At*bZIP63, *At*WRKY11^DBD^) were expressed in *E. coli *BL21/RIL cells and extracted under denaturing conditions. Renaturing and refolding were performed by dialysis over night in either native buffer (50 mM Tris/HCl pH 8; 100 mM NaCl) or directly in protein extraction buffer. Binding reaction was performed with different concentrations of protein, 1 μg poly(dI-dC) and 2 ng of ^33^P-radioactively labelled DNA probe. DNA-protein complexes were separated from unbound probes on native 6% polyacrylamide gels, or 3% polyacrylamide gels supplemented with 0.6% agarose, in TBE buffer at 200 V for 2.5 hours. After electrophoresis, the gels were dried and subjected to autoradiography at -70°C with an intensifying screen.

### Recommended buffers and equipment for DPI-ELISA

#### Ds-bio DNA probe

for short sequences biotinylated (sense) and non-biotinylated (antisense) oligonucleotides were ordered from companies (Metabion, Biozym); for long sequences PCR with one 5' biotinylated primer can be used [9]

#### Annealing buffer

40 mM Tris-HCl pH 7.5-8, 20 mM MgCl_2_, 50 mM NaCl

***Protein dilution buffer ***(is equivalent to protein extraction buffer without proteinase inhibitors)

4 mM HEPES pH 7.5, 100 mM KCl, 8% (v/v) glycerol, 0.2% biotin-free BSA, 5 mM dithiothreithol (DTT)

#### TBS-T

20 mM Tris-HCl pH7.5, 180 mM NaCl, 0.1% (v/v) Tween20

#### PBS

10 mM Na_2_HPO_4_/NaH_2_PO_4 _pH7.5, 140 mM NaCl

#### PBS-T

10 mM Na_2_HPO_4_/NaH_2_PO_4 _pH7.5, 140 mM NaCl, 0.1% (v/v) Tween20

#### Blocking reagent

5% non-fat dry milk (Roth) in TBS-T or antibody specific blocking reagent according to the manufacturers' instructions (

-His:HRP antibody specific from Qiagen)

#### Antibody

α-epitope specific antibody conjugated with horseradish peroxidase (α-His:HRP from Qiagen 1:1'000 in PBS-T) or α-protein specific primary antibody and secondary antibody conjugated with horseradish peroxidase [9]

#### OPD-solution

4 mg *ortho*-phenylenediamine (OPD-tablets from Sigma), 3 μl 30% H_2_O_2 _in 6 ml CP-buffer

#### CP-buffer

10 mM Na_2_HPO_4_, 100 mM citric acid, pH 5 with NaOH

#### Stopping solution

2N HCl

#### ELISA micro well plates

streptavidin-coated (5 pmol/well), pre-blocked, clear 96-well plates (Reacti-Bind™streptavidin coated clear 96-well plates with SuperBlock blocking buffer from Pierce, Thermo Fisher Scientific)

#### ELISA-reader

Tecan Safire plate reader

### Detailed protocol of the DPI-ELISA

**Figure 1:**
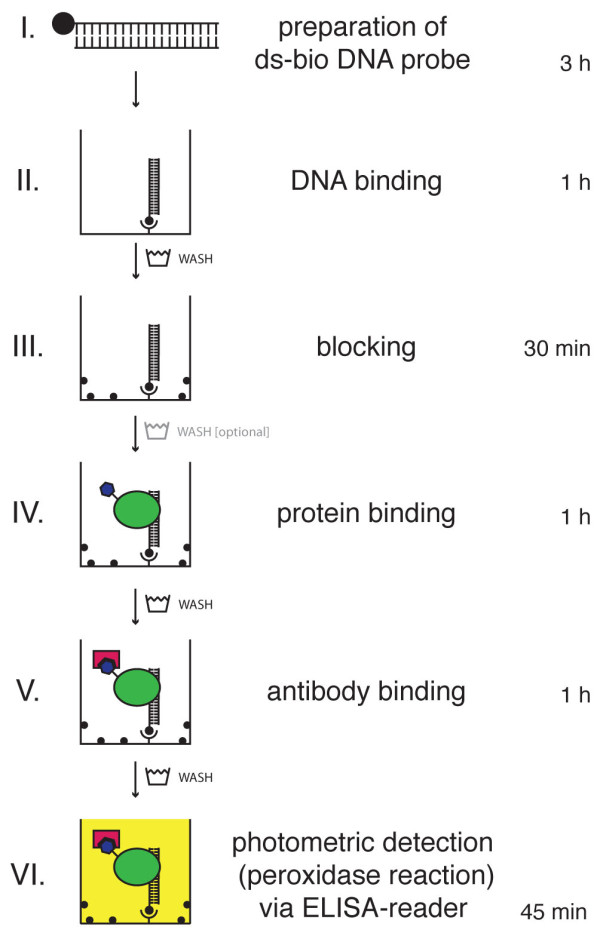
**Schematic workflow of the DPI-ELISA**. Double stranded biotinylated (ds-bio) DNA-probes (I) are immobilised on a streptavidin-coated microtiter plate (II). After blocking the plate with an appropriate reagent (III), incubation with a crude protein extract from *E. coli *containing the epitope tagged protein under investigation is performed (IV). The epitope tagged protein is retained inside the well by physical binding to the immobilized DNA and, thus, can be detected with appropriate antibodies - which are conjugated with horseradish peroxidase in our experiments (V). Finally, peroxidase substrate (OPD) is added for the colorimetric quantification of specifically bound transcription factors to dsDNA-probes (VI). Between each of the workflow steps, at least three washing steps of the microtiter plate are performed; washing between steps III and IV is optional. Incubation times and approximate duration of the photometric detection step (peroxidase reaction) are given at the right hand side.

The overview of the entire protocol is displayed in Figure 1. All steps are carried out at room temperature, except step II (37°C). Careful washing between each step is recommended.

*I Preparation of ds-bio DNA probe *{3 hours}:

Short single stranded DNA (25-30 nucleotides) can be ordered from various companies; we ordered non-modified oligonucleotides (Metabion) and 5' biotinylated complementary oligonucleotides (Biozym) independently. The sense and antisense oligonucleotides were diluted in annealing buffer (2 μM each) and heated in a water bath for 3 min at 95°C and left to cool down slowly to room temperature to construct the ds-bio DNA probe (2 μM). If longer ds-bio DNA fragments are desired, PCR with one 5' biotinylated primer is recommended [9]. The ds-bio DNA probe was stored at -20°C.

*II DNA-binding to the streptavidin-coated plates *{1 hour}:

The most informative results were gained with 2 pmol of ds-bio DNA in TBS-T added to each well and incubated at 37°C (60 μl per well).

Wash: 3 × 150 μl TBS-T

*III Blocking of residual binding spots of the micro well plate *{30 min.}:

Blocking was performed with either 5% non-fat dry milk (Roth) in TBS-T or α-His:HRP antibody specific blocking reagent (Qiagen) (100 μl per well).

Wash: 3 × 150 μl TBS-T (optional, but recommended)

*IV Protein binding to immobilised ds-bio DNA *{1 hour}:

Up to five different amounts of total protein extract in protein dilution buffer should be tested (e.g. 0.5 μg, 5 μg, 25 μg, 50 μg, 100 μg in 60 μl per well).

Wash: 3 × 150 μl PBS-T

*V Antibody binding of protein bound to DNA *{1 hour}

We recommend using an antibody that is directly conjugated with horseradish peroxidase for the immunological detection of the α-epitope. We used α-His-HRP antibody conjugate (Qiagen) diluted 1:1'000 in PBS-T (60 μl per well).

Wash: 2 × 150 μl PBS-T, 2 × 150 μl PBS.

*VI Photometric detection (peroxidase reaction) via ELISA-reader *{< 45 min.}: Plates were incubated with OPD-solution (60 μl per well) in the dark for max. 30 minutes. After adding an equal volume of stopping solution, the plate was kept for further 10 minutes in the dark under mild agitation (150 rpm). The extinction was measured at 492 nm using 650 nm (plate background) as a reference wavelength in the ELISA-reader. In the case of kinetic measurements no stopping solution was added and the absorbance was measured at 450 nm for 1 hour with an interval of 5 min.

### Data analysis

**Figure 2:**
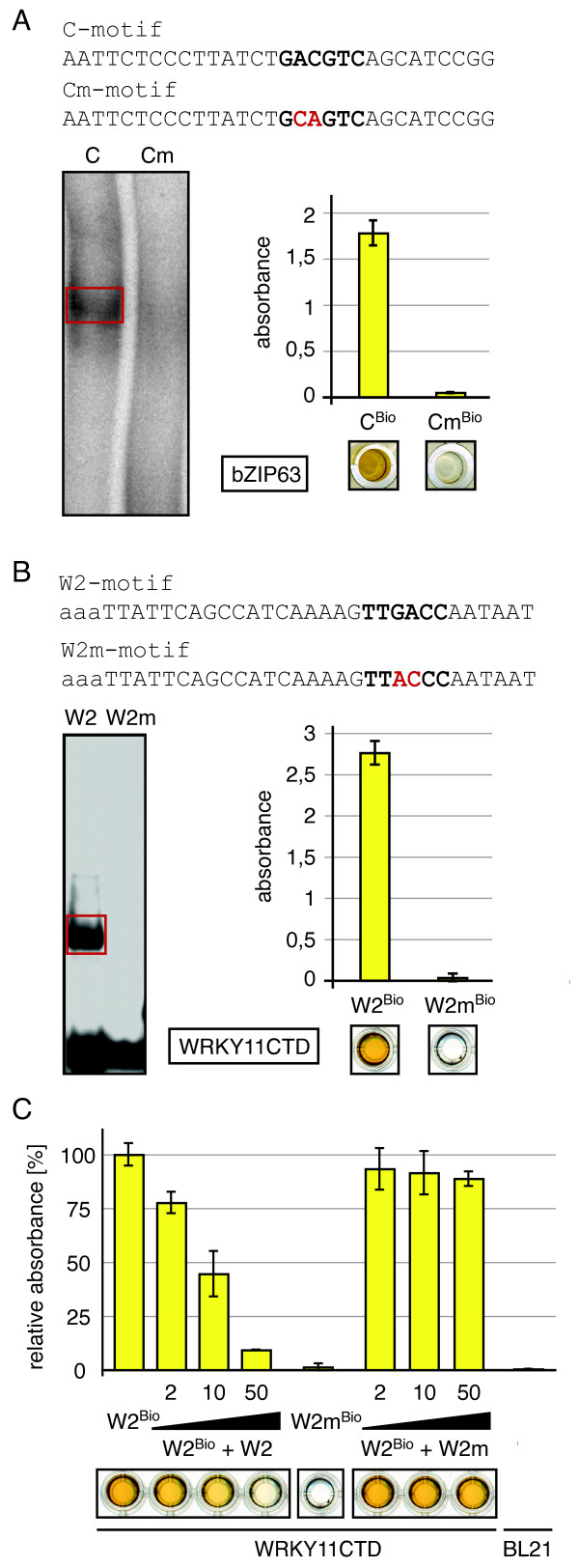
**Comparison of the classical EMSA and the DPI-ELISA**. The specific binding to DNA is investigated with plant transcription factors of two classes: *At*bZIP63 (**A**.) and *At*WRKY11^DBD ^(**B**.). Specific binding of the recombinant proteins to double stranded (ds) DNA-probes by electrophoretic mobility shift assays (EMSA; left panel) or DPI-ELISA (right panel) is displayed. The sequences (top panel) of the dsDNA-probes are given in 5'-3'-orientation for the sense strand; changes of bases within the known binding consensi (bold face) are highlighted (red) in the mutated oligonucleotide versions [13,14]. For EMSA, specific retardation bands are highlighted by red boxes; for the DPI-ELISA a picture of the respective plate-wells is displayed below each column of the histogram graph. **A**. Renatured *At*bZIP63 is tested with double stranded C- and Cm-probes. **B**. *At*WRKY11^DBD ^contained in crude protein extract from *E. coli *is tested with double stranded W2- and W2m-probes. Both experiments (**A**. + **B**.) confirm results from previous publications [13,14]. **C**. Competition experiment: The specific binding of *At*WRKY11^DBD ^to W2-probes is competed with non-biotinylated dsDNA. Different amounts of W2- or W2m-probe (0, 2, 10, 50 pmol) were added to *At*WRKY11^DBD ^crude extract immediately prior the plate incubation. ELISA-plates are coated with 2 pmol of double stranded biotinylated W2^Bio^-probe. The biotinylated dsDNA W2m^Bio^-probe incubated with *At*WRKY11^DBD ^extract or the W2^Bio^-probe incubated with BL21/RIL cells (transformed with an empty vector construct) serve as negative control.

**Figure 3:**
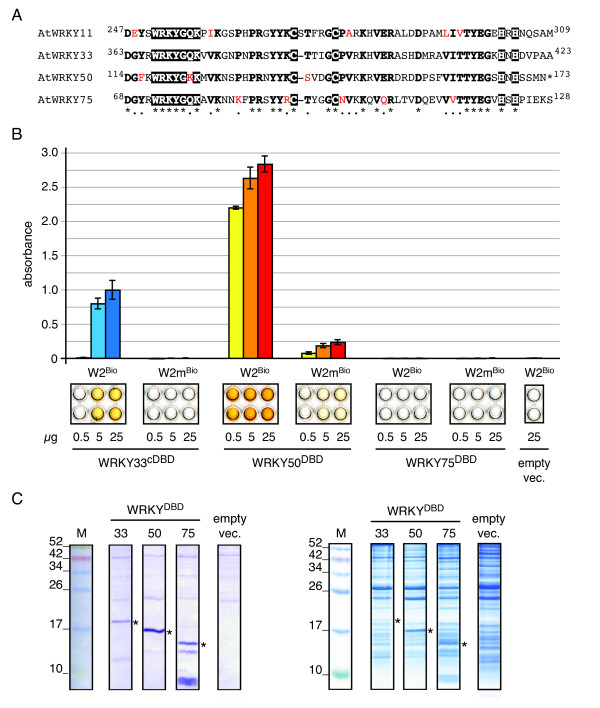
**DNA-binding capacity of *Arabidopsis thaliana *WRKY33^cDBD^, WRKY50^DBD ^and WRKY75^DBD ^to the W2-probe**. **A**. Amino acid alignment of WRKY11, WRKY33, WRKY50 and WRKY75 DNA-binding domain (DBD) sequences. The highly conserved WRKY-consensus and the zinc-finger are highlighted (white on black); conserved amino acid residues are displayed in bold face. Non-conserved residues that might contribute to differences in WRKY-domain function by altering the binding specificities are highlighted in red. **B**. DPI-ELISA results for *At*WRKY33^cDBD^, *At*WRKY50^DBD ^and *At*WRKY75^DBD ^binding to the W2- or W2m-probes. Different amounts of extracts (0.5, 5, 25 μg total protein per well) were examined with W2^Bio^- and W2m^Bio^-probes. Representative wells of the microtiter plate are shown below the graph for visual inspection. **C**. Detection of the immobilized His-epitope tagged proteins with anti-His-antibodies in the crude extract by western blotting using (left). Asterisks indicate the appropriate bands (*At*WRKY33^cDBD ^- 20 kDa, *At*WRKY50^DBD ^- 17 kDa, *At*WRKY75^DBD ^- 15 kDa). Coomassie-stained SDS-PAGE (right) is shown for equal loading of the gel.

The *absorbance *values displayed are the mean of two independent samples and standard deviation (Figure 2A and 2B, 3B).

The *relative absorbance *data provides the mean of two independent samples and standard deviation, relative (in percentage) to the *At*WRKY11^DBD ^- W2^Bio^-probe mean (Figure 2C).

**Figure 4:**
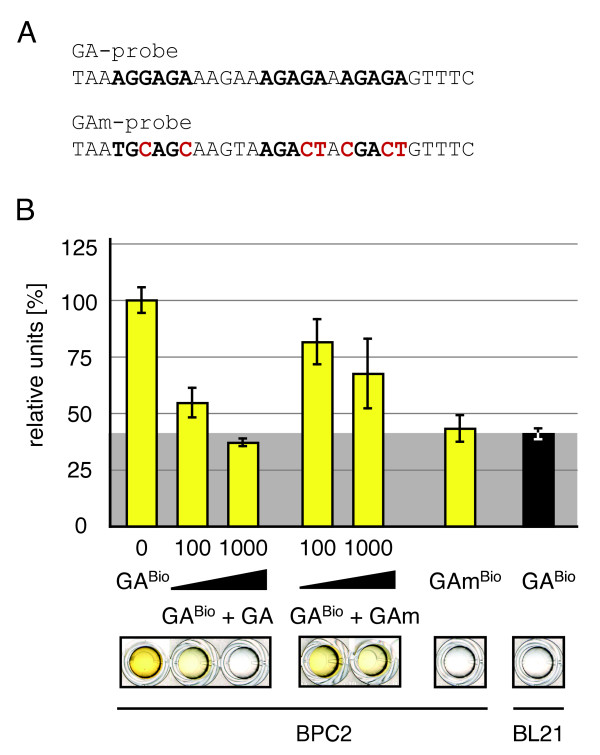
**Employing the DPI-ELISA to other transcription factors**. **A**. The DNA-sequences of the double stranded GA-probe and the mutated oligonucleotide version (GAm-probe) are given in 5' to 3'-orientation for the sense strand; mutated bases are highlighted (red) [25]. The specific binding of *At*BPC2 to the GA^Bio^-probe was shown by a competition experiment with non-biotinylated dsDNA (**B**.). Different amounts of GA- or GAm-probes (0, 100, 1000 pmol) were added to *At*BCP2 crude extract and incubated on an ELISA-plate coated with 2 pmol of biotinylated GA^Bio^-probe. The biotinylated double stranded GAm^Bio^-probe incubated with *At*BPC2 extract served as negative control. Representative wells of the microtiter plate are shown below the graph for visual inspection. The grey background indicates the negative control (untransformed BL21/RIL cells) reference values in percent.

The *relative units *data was calculated by normalisation of the mean of two independent samples and standard deviation to the BL21 negative control (Figure 4A). The fold differences of the negative interaction (*At*BPC2 - GAm-probe) to positive interaction (*At*BPC2 - GA-probe) were calculated after subtraction of the BL21 negative control (1 rel. unit) (Figure 4B).

**Figure 5:**
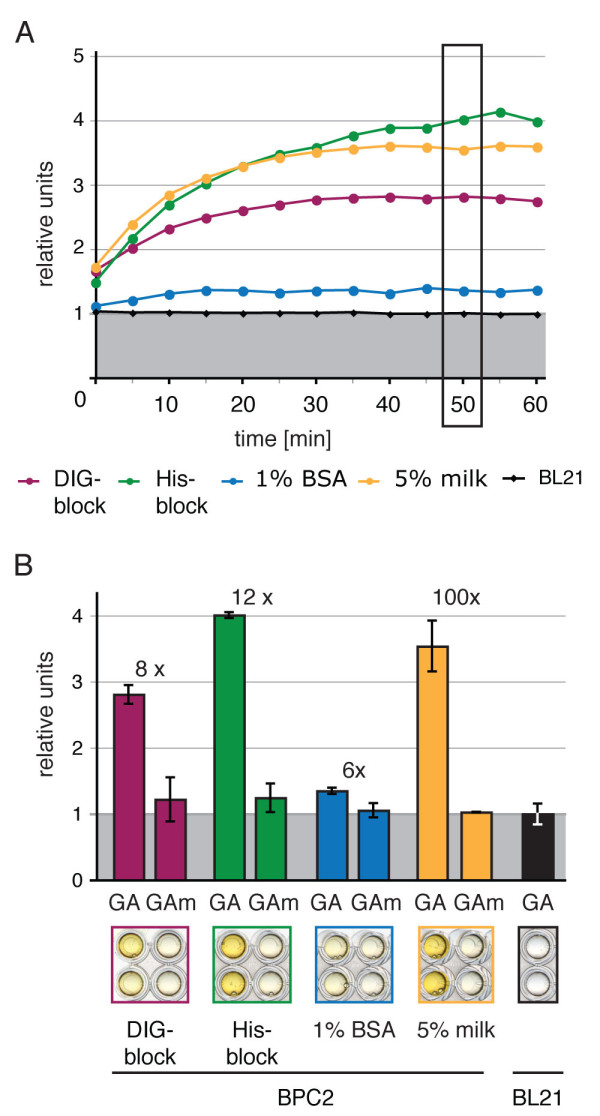
**The absorbance of positive DNA-protein-interaction is affected by blocking reagents**. Changes in binding affinity and fold differences between positive and negative DNA-protein interaction for different blocking reagents are analysed with *At*BPC2 and GA- or GAm-probes (**A**. + **B**.): α-DIG blocking reagent (DIG-block), α-His blocking reagent (His-block), 1% bovine serum albumin (BSA) in TBS-T and 5% non-fat dried milk in TBS-T (milk). **A**. The average OPD-turnover is measured in an experiment over 60 min. **B**. The normalised values and standard deviations at 50 minutes incubation time are graphed as histograms for the four different blocking reagents. The background-normalised fold-differences are given above the respective columns. Representative wells of the microtiter plate are shown below the graph for visual inspection. The grey background indicates the negative control (untransformed BL21/RIL cells) reference values.

In cases in which data of two ELISA-plates was joined (Figure 5B), the mean of two independent samples was divided by the plate specific mean of the BL21 negative control, leading to *relative units*. Next, the respective means and standard deviations of both ELISA-plate data sets were calculated. Finally, the unified *relative unit*s data is given relative (in percentage) to the *At*BPC2 - GA^Bio^-probe mean.

## Results and Discussion

### Comparison of EMSA and DPI-ELISA to elucidate DNA-protein interaction

Initially, we asked whether the results obtained by our DPI-ELISA protocol (Figure 1) are qualitatively comparable to those from well-established methods like EMSA. We chose to study *At*bZIP63, as two previous publications utilised a similar ELISA-based transcription factor assays for the analyses of *Arabidopsis thaliana *bZIP transcription factors but did not show a comparison to EMSA data [12,13]. We analysed the DNA-binding specificities of the same protein extract of *At*bZIP63 to the C-box by both, EMSA and DPI-ELISA (Figure 2A). Both methods resulted in the same assertion, that *At*bZIP63 is capable of binding to the C-box containing probe [13], but not to its mutated version (Cm-probe).

We next used the DPI-ELISA on a selected member of the WRKY transcription factor family from *Arabidopsis*. Ciolkowski et *al*. [14] already investigated the binding sequence of some *At*WRKY proteins by EMSA, which they found to be the W2-box for all tested WRKY family members. We decided to verify the binding specificity of the DNA-binding domain of *At*WRKY11 (*At*WRKY11^DBD^) by EMSA and DPI-ELISA (Figure 2B). For this EMSA crude *E. coli *extracts were used, which were extracted under denaturing conditions and subjected to renaturation and refolding steps; for the DPI-ELISA proteins were extracted under native conditions. Indeed, both methods revealed that *At*WRKY11^DBD ^specifically bound to the W2-box but not to its mutated version (W2m-probe) (Figure 2B). To define the specificity of the DNA-protein interaction by DPI-ELISA, we competed the *At*WRKY11^DBD ^- W2^Bio^-probe interaction by adding varying amounts of non-biotinylated W2-probe to the protein binding reaction (Figure 2C). We could show that an equal amount of biotinylated versus non-biotinylated W2-probe already decreased the absorbance by 25%. Additionally, increasing amounts of non-biotinylated W2-probes successfully competed with the binding of *At*WRKY11^DBD ^to the immobilized biotinylated W2-probe in a concentration dependent manner. Finally, the addition of 50 pmol W2-probe almost abolished the DNA-protein interaction. In contrast, the interaction between *At*WRKY11^DBD ^and the W2-probe could not be competed with varying amounts of non-biotinylated W2m-probe, which verified the sequence specific *At*WRKY11^DBD ^- W2-box interaction (Figure 2C). These results clearly demonstrate that the DPI-ELISA is a valuable method for the analysis of DNA-protein interactions and that the results were comparable to those of the classical EMSA. Additionally, we could show that the DNA-binding domain of a transcription factor is sufficient for the study of DNA-binding specificities by DPI-ELISA, which is especially important in cases of cellular lethality caused by full-length proteins [14,17].

### Application of the DPI-ELISA to other WRKY-proteins from *Arabidopsis thaliana*

The plant specific WRKY transcription factors constitute a very large subgroup of the WRKY-GCM1-superfamily of zinc-finger DNA-binding proteins that are variable in length and can be composed of diverse domains. Common to all family members is the highly conserved WRKY-domain, which mediates DNA-binding and, hence, confers the specificity of the DNA-protein interaction [18,19]. The analysis of the domain composition and phylogenetic investigations of the conserved WRKY-domain sequence led to the sorting of the proteins into several (sub-) groups. The high sequence similarity of the WRKY-domain and the results of previous experiments led to the speculation that all WRKY-proteins might possibly recognise the same consensus, which would be the W-box (5'-TTGACC/T-3') [14,18,20,21].

For further studies we chose three WRKY-transcription factors from different groups: *At*WRKY33 constitutes a group I member and is the *Arabidopsis thaliana *ortholog of the well-studied *Pc*WRKY1 of parsley [22-24]. Proteins from this WRKY-group have two WRKY-domains, of which only the C-terminal DNA-binding domain (cDBD) has been shown to mediate DNA-binding [14,18]. *At*WRKY50 and *At*WRKY75 belong to subgroup IIc, which forms an independent phylogenetic clade in WRKY-domain analyses, that has not been in the focus of research so far [18,20,21]. The conserved WRKY-domains of both proteins are only a little shorter in size than the full-length proteins [18] and disclose several changes at otherwise conserved amino acid positions (Figure 3A). Interestingly, *At*WRKY50 possesses an aberrant WRKYG**K**K peptide-motif and, thus, lacks the almost invariant WRKYG**Q**K consensus, which possibly confers the specific binding to the 5'-TGAC-3' W-box core. However, there are only subtle amino acid differences between the WRKY-DNA-binding domains (DBDs) of *At*WRKY11, *At*WRKY33, *At*WRKY50 and *At*WRKY75 in general (Figure 3A).

As we were interested in whether the chosen WRKY-proteins do all bind to the W2-box, we tested these interactions by DPI-ELISA. Indeed, *At*WRKY33^cDBD ^and *At*WRKY50^DBD ^bound to the W2-probe (Figure 3B). The absorbance of *At*WRKY50^DBD ^- W2-probe was more than 2-fold higher than *At*WRKY33^cDBD ^- W2-probe. This can be partially explained by different concentrations of the epitope tagged WRKY-protein within the crude protein extract, which could be supported by the western transfer (Figure 3C). Another reason for the differences in the absorbance of *At*WRKY50^DBD ^- W2-probe and *At*WRKY33^cDBD ^- W2-probe could be a promiscuous binding to a more degenerate DNA-sequence consensus. Interestingly, *At*WRKY50^DBD ^also exhibited a weak affinity to the mutated W2m-probe, which was not observed for *At*WRKY11^DBD ^or *At*WRKY33^cDBD^, even when higher protein amounts were added (data not shown). We assume that these differences in binding specificity directly translate to altered residues in the DNA-binding domain (Figure 3A). The most apparent difference is the glutamine to lysine exchange within the WRKYG**Q**K consensus of *At*WRKY50 (WRKYG**K**K), in which the positively charged lysine might be responsible for the increased affinity to the W2m-probe.

In contrast to our findings on *At*WRKY50^DBD^, *At*WRKY33^cDBD ^or *At*WRKY11^DBD^, we could not detect any binding of *At*WRKY75^DBD ^to the W2- or the W2m-probe, even when 100 μg of crude protein extract were loaded (data not shown). As *At*WRKY75^DBD ^was present at the expected size on the SDS-PADE with subsequent western analysis and in equivalent amounts compared to the other tested WRKY-proteins, we assume that *At*WRKY75^DBD ^did not recognise any binding motif inside the respective DNA-probe sequences.

### Application and adjustment of DPI-ELISA to another *Arabidopsis thaliana *transcription factor - Basic Pentacysteine 2

To elucidate the applicability of the DPI-ELISA, we applied the method to *Arabidopsis *Basic Pentacysteine 2 (*At*BPC2), a zinc finger-like transcription factor, which had not been studied so far. *At*BPC2 constitutes a putative GA/TC-repeat binding protein and is a close homolog of *At*BPC1, that has already been shown to bind a DNA-consensus of the GA-probe [25,26]. During first experiments we could indeed show that *At*BPC2 is capable of binding to the GA-probe but not to the GAm-probe, where the specific GA/TC-repeat binding sites were mutated (Figure 4). To test the binding specificity of the *At*BPC2 - GA-probe interaction, the binding was competed with increasing amounts of non-biotinylated double stranded GA- or GAm-probe (Figure 4B). The addition of the GA-probe to the protein binding of *At*BPC2 specifically prevents positive interaction with the immobilised biotinylated GA-probes in a concentration dependent manner, while this binding could not be competed with the GAm-probe (*see also *trouble shooting). The addition of 1000 pmol DNA generally reduced the protein binding specificity irrespective of the DNA-sequences, which may be the result of a concentration dependent sterical hindrance of the protein-DNA interaction.

A crucial variable of the protocol, which could significantly interfere with specific DNA-protein interaction, is the blocking of the wells after the DNA immobilisation. To provide insight into the possible outcomes, the effect of four frequently used blocking reagents was tested on the *At*BPC2 interaction with the GA- or GAm-probe (Figure 5). The highest fold differences of the signal over the background were achieved with the His-specific blocking reagent (His-block from Qiagen) and with 5% non-fat dried milk (milk). In contrast, the fold difference and the normalised absorbance were lowest with the DIG-specific blocking reagent (DIG-block, Roche) or with 1% bovine serum albumin (BSA). This might be due to unspecific protein-protein interaction or an elevated biotin amount, which is capable of competing with the ds-bio DNA-probes for the streptavidin coated sites within the wells. We tested the effect of increasing amounts of biotin and found a concentration dependent decrease of the positive interaction signal (data not shown). Although milk - like BSA-contains an unknown amount of naturally occurring biotin, the highest fold differences were gained with 5% non-fat dried milk (milk). In conclusion, we recommend both the His-specific blocking reagent and milk for blocking.

### Assessing the linear absorbance range for quantitative measurements

**Figure 6:**
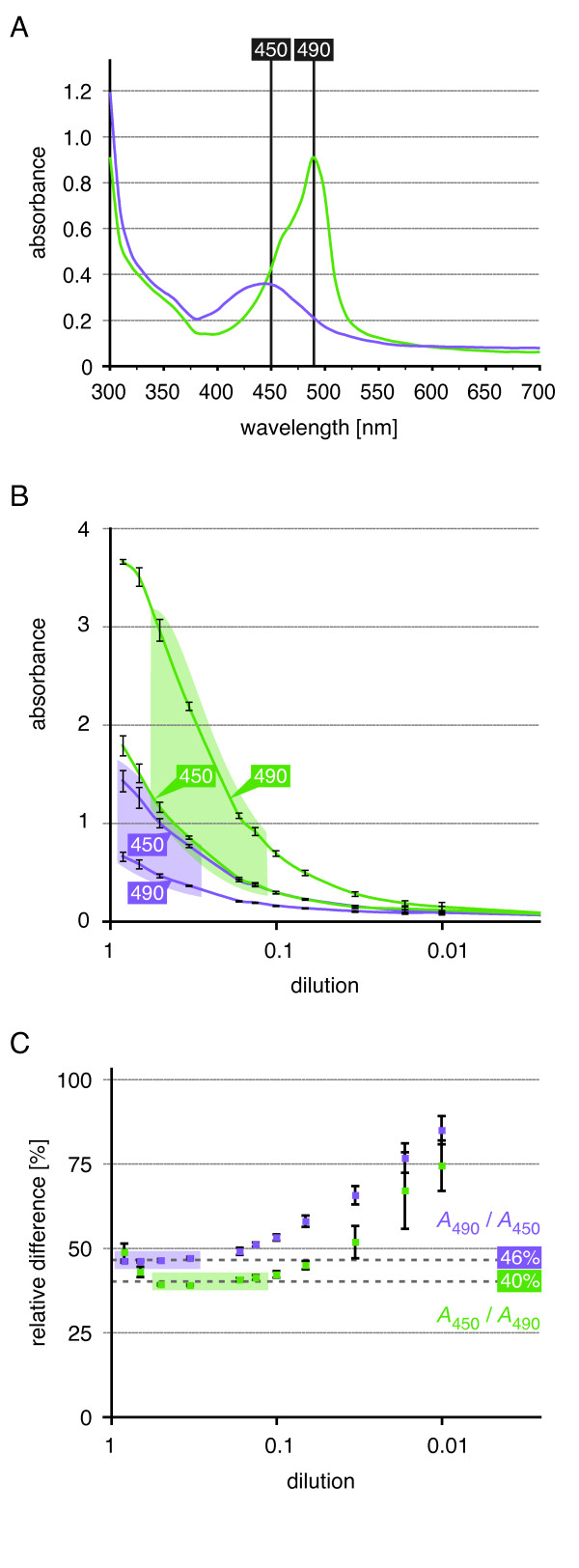
**Influence of measurement wavelength on absorbance values**. **A**. The OPD-solution at pH 5 shows the highest maximum absorbance at 450 nm (purple curve). The peroxidase reaction is stopped for end-point measurements by acidification (addition of stopping solution), which leads to a shift in the absorbance spectrum with an absorbance maximum at 490 nm (green curve). **B**. + **C**. Dilution series of OPD-reaction product in CP-buffer is displayed along the x-axis [log scale]. Concentration dependent differences in the absorbance values measured at 450 nm and 490 nm before (purple lines) and after the addition of stopping solution (green lines). **B**. Range of linear relationship between the measurements at the two wavelengths is highlighted by coloured backgrounds. **C**. The relative difference between the measurements of the stopped solution [*A*_450_/*A*_490_] is ~40%, while it is ~46% for the non-stopped reaction [*A*_490_/*A*_450_].

One advantage of the DPI-ELISA is the opportunity to quantify the results by photometry. However, the absorbance spectrum of the OPD-reaction product is pH dependent (Figure 6A). The absorbance maximum of the OPD-reaction product in CP-buffer (pH 5) lies at about 450 nm, whereas after addition of stopping solution (HCl) the pH decreases, which results in a shift of the absorbance spectrum to an absorbance maximum close to 490 nm [27]. Qualitative measurements should be performed always within the absorbance maximum, but for quantitative measurements it is necessary to obtain absorbance data within the linear absorbance range of the photometer. This range is dependent on the sensitivity of the detector within the ELISA-reader. To define the linear absorbance range of the ELISA-reader, we performed a dilution experiment with the OPD-reaction product (Figure 6B). The absorbance of the dilution series was analysed before and after the addition of stopping solution at both 450 nm and 490 nm, which measures either in the border region of the two spectra or at their absorbance maxima, respectively. Subsequently, the relative difference can be calculated by division of the absorbance in the boarder region by the absorbance at the maximum (Figure 6C). This finally leads to the definition of the linear absorbance range of the ELISA-reader: Before acidification the limit of the linear absorbance range can be assigned by the relative difference of *A*_490_/*A*_450_. In our experiments all values above 0.36 have the constant relative difference of 46%. After acidification the values for the higher and the lower limits of the linear absorbance range lay between 0.38 and 3.0 with the constant relative difference of 40% (*A*_450_/*A*_490_). This analysis (Figure 6B + 6C) also allows the correction of data obtained outside the linear range for qualitative measurements.

In conclusion, it is necessary to define the linear absorbance range of each ELISA-reader to obtain quantitative data. Therefore, all absorbance data between 0.36 and 3.0 allows the quantitative readout with our ELISA-reader. For quantitative experiment we recommend measurements at both, the absorbance maximum and the border region of the spectrum.

### Trouble-shooting

**Figure 7:**
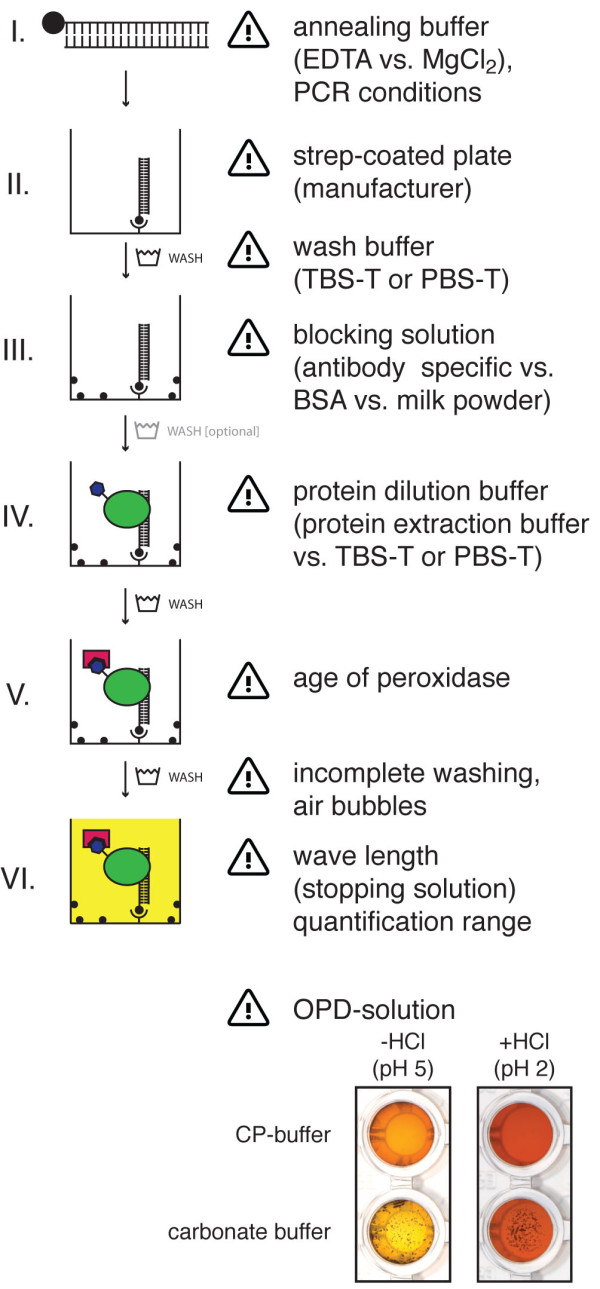
**Notes for trouble-shooting**. The figure provides an overview of crucial points of the DPI-ELISA and notes for trouble-shooting. At the bottom of the panel the effects of different OPD-solution buffers are shown: A carbonate buffer will lead to the precipitation of the coloured OPD-reaction product, if it accumulates to high amounts. Interestingly the sediment dissolves slowly after stopping the reaction with hydrochloric acid (data not shown). In contrast, a transparent solution is obtained with the CP-buffer before and after addition of stopping solution.

Although the DPI-ELISA is fast and simple compared to other methods, there are some aspects that need to be noted for trouble-shooting (Figure 7):

I Annealing buffer for the ds-bio DNA-probes:

It is of general importance to balance reasons for using EDTA or EGTA to neutralise DNases by forming complexes with Mg^2+ ^*versus *an unwanted counteraction of the DNA-protein interaction. We recommend using chelate-free buffers.

II Strep-coated plate:

Most standard capacity streptavidin-coated plates were coated with 5 pmol streptavidin per well. We recommend using pre-blocked plates, as the data variation was smaller. Pre-blocked plates exhibited a lower background compared to non-pre-blocked ones. Nevertheless, additional blocking after the ds-bio DNA immobilisation is still advisable and could further decrease background signals. We ascertained that 2 pmol of ds-bio DNA-probes are sufficient to obtain optimal signals, as described before [9]. It is also possible to coat the plates with streptavidin (5 pmol) in one's own lab, which extends the protocol duration by one day.

Wash: Our experience showed that it is important to take particular care during the wash procedures in general. In some cases, e.g. with *At*bZIP proteins it is better to use only PBS-T for washing, as the absorbance values were significantly higher than those after washing with TBS-T.

III Blocking solution:

We recommend using an antibody specific blocking reagent if available. For the majority of the experiments blocking with non-fat dried milk is recommendable.

Wash [optional]: We found that this washing step can be omitted in most of the experiments, as no changes in signal intensities were observed.

IV Protein dilution buffer:

We recommend using the protein extraction buffer without additional proteinase inhibitors. The proteins are stable and soluble within this buffer; as suitable alternatives one could use TBS-T or PBS-T. The decision for or against a certain buffer for protein dilution or extraction is critical as these are variable and account for the specific needs attributed to the biochemical characteristics of the DNA-binding proteins. For example: the redox-state of disulfide bonds, ion chelate formation in zinc finger transcription factors or the phosphorylation state of the protein needs to be considered for buffer composition and, hence, might affect DNA-binding. We recommend using a HEPES-buffer for extraction, as this is the most commonly used one (Additional file 1).

NOTE: *When expressing epitope-tagged proteins in *E. coli, *the formation of inclusion bodies can take place resulting in lesser amount of native protein. In general it is recommended to add proteinase inhibitors to the extraction buffer to slow down protein degradation and to consume protein extracts quickly*.

NOTE: *A successful competition experiment is performed best at a protein concentration that yields half-maximal signal intensities. Therefore, different amounts of crude *E. coli *protein extract (e.g. 0.5 - 100 μg) need to be tested. The subsequent addition of 2 pmol, 10 pmol and 50 pmol non-biotinylated DNA-probes should then be sufficient to fully compete with the positive DNA-protein interaction*.

V Age of peroxidase:

It is important to notice, that protein degradation of antibody conjugated horseradish peroxidase occurs and, moreover, that the enzyme activity of the horseradish peroxidase is reduced over time respective to the buffer conditions [27].

Wash: Incomplete washing before the enzymatic reaction leads to an increased background signal and, thus, complete discharging of the plate (e.g. by firm up-side down shaking) is recommended after washing to remove as much of the residual liquid as possible. Cautious pipetting is needed, especially at this step, to avoid bubbles that disturb the subsequent measurements.

VI OPD-solution and notes for measurement:

We experienced a dependency of the solubility of the coloured OPD-reaction product on the buffer system (see Figure 7 bottom). We obtained the best results using a phosphate-citrate buffer with 10 mM phosphate, as higher phosphate concentrations inhibit the peroxidase irreversibly [27]. In contrast, the use of a carbonate buffer caused precipitation of the product that lead to a reduced absorbance.

To obtain qualitative results, we recommend measurements at the absorbance maximum. Quantitative evaluations can be conducted within the linear absorbance range of the ELISA-reader (see Figure 6).

*NOTE: The quality of the solutions, antibodies and plates can be monitored by the use of appropriate controls (e.g. without immobilised ds-bio DNA, without the addition of protein extracts, etc.)*.

*NOTE: Another appropriate stopping solution is 2N H_2_SO_4_*.

## Conclusions

The DPI-ELISA comprises a fast, non-radioactive, easy to use and cost-efficient method to study the interaction between plant transcription factors and DNA-probes. We could show that this method yields results comparable to EMSA with both the full-length proteins and the DNA-binding domains of plant transcription factors, only. Previous results with DPI-ELISA on human NFκB demonstrated its high sensitivity and robustness [7,9,10]. Our data on plant transcription factors were fully in accord with these previous findings and, hence, it can be concluded that DPI-ELISA with plant DNA-binding proteins should be 10-times more sensitive than the traditional EMSA [8,9]. In contrast to EMSA, DPI-ELISA offers the opportunity to investigate the binding specificities of proteins to DNA of any length and, thus, long promoter sequences and short DNA-binding sequences can easily be studied side-by-side on one plate.

## Competing interests

The authors declare that they have no competing interests.

## Authors' contributions

LHB and DW conceived and designed the experiments. LHB TK SH CC DW performed the experiments and analysed the data. LHB CC DW wrote the manuscript.

All authors have read and approved the final manuscript.

## Supplementary Material

Additional file 1 **Comparison of five previously published DPI-ELISA protocols**. Table summarising the main points of each publication corresponding to research focus, protein expression and extraction, DNA preparation, DPI-ELISA procedure and conclusive results. Selected were the five research publications that report the respective protocol for the first time - all of which contributed to human inflammatory research [6-10]. Click here for file
