# Supplementary material for: DPI-ELISA: a fast and versatile method to specify the binding of plant transcription factors to DNA in vitro
Source: Plant Methods. 2010 Nov 25;6:25. doi: 10.1186/1746-4811-6-25 (PMC3003642; doi:10.1186/1746-4811-6-25)
Supplement: Additional file 1 — Comparison of five previously published DPI-ELISA protocols. Table summarising the main points of each publication corresponding to research focus, protein expression and extraction, DNA preparation, DPI-ELISA procedure and conclusive results. Selected were the five research publications that report the respective protocol for the first time - all of which contributed to human inflammatory research [6-10]. [file 1746-4811-6-25-S1.PDF]

	Brand et al. (this publication)	Jagelská et al. (2001)	Renard et al. (2000)	Benotmane et al. (1997)	Gubler and Abarzúa (1995)	Hibma, Ely and Crawford (1994)
**Topic**	*Arabidopsis thaliana* WRKY11, WRKY50, WRKY33, bZIP63, BPC2 binding to DNA	human p53 tumor supressor protein	tested different settings of the ELISA-procedure with purified NFkB and crude-cell extracts	human 6D3 fragment of HLTF, quantification of protein-DNA interaction at equilibrium, detection of [1] DNA bound to immobilised protein vs. [2] protein retained by immobilised DNA	human p53 binding to DNA, crude- vs. purified extracts, temperature-sensitive p53 mutant	active human papillomavirus type (HPV 16) E2 protein binding to DNA
**Protein Expression**	pET-DEST42 (Invitrogen) in *E. coli*, induction with IPTG	mCF-7 human tumor cell line, induction with UV light	human fibroblast cells		Saos-2, Cos cells; 1h induction with 10^{8}M b-estradiol	pGEX - GST:HPV16 E2 in *E. coli*, induction with IPTG
**Protein Extraction**	sonification; control pET-DEST42 no insert; extraction buffer (4 mM HEPES, 100 mM KCl, 8% glycerol, 0.2% BSA without Biotin, 5 mM DTT, proteinase inhibitors without EDTA (Roche))	extraction buffer (20 mM HEPES pH 7.6, 0.5M NaCl, 20% glycerol, 10 mM NaCl, 1.5 mM $MgCl_2$, 0.2 mM EDTA pH 8.0, 0.1% NP-40, 1 mM DTT, protease and phosphatase inhibitors)	extraction buffer (20 mM HEPES pH7.5, 0.35 M NaCl, 20% glycerol, 1%NP-40, 1 mM $MgCl_2$*$6H_2O$, 0.5 mM EDTA, 0.1 mM EGTA, proteinase inhibitor)	sonification; extraction buffer (150 mM NaCl, 16 mM Na_2HPO_4, 4 mM NaH_2PO_4, < 1% TritonX-100); purification	freezing; extraction buffer (20mm HEPES pH7.9, 1 mM DTT, 1mM EDTA, 1mM EGTA, 0.4M KCl, 1mM PMSF, 1µg/ml leucopeptine, 1µg/ml aprotinin, 1µg/ml pepstatin, 20% glycerol)	sonification; control pGEX no insert; binding and extraction buffer (40mM Tris-HCl pH8, 140mM KCl, 4mM $MgCl_2$, 40µM zinc acetate, 0.4 mM DTT, 12% glycerol, 0.2% Tween20, 4%BSA, 40µg/ml calf thymus DNA); purification
**DNA Preparation**	~30 bp long DNA; 5' end biotinylation in annealing buffer (40 mM Tris/HCl pH 7.5, 20 mM $MgCl_2$, 50 mM NaCl); 2 pmol ds-bio DNA/well in TBS-T	annealing of 20- 58 bp long DNA, 5' end biotinylation; 500 ng ds-bio DNA per reaction	122 bp long DNA by PCR, 5' end biotinylation 2 pmol ds-bio DNA/well in PBS + 0.1% Tween20; quantification of fixed DNA by Picogreen Assay (Molecular Probes, OR)	annealing of 27 bp long DNA (B-box) in annealing buffer (10 mM Tris-HCl pH7.5, 0.1 M NaCl); 5' end biotinylation: [1] 0-200nM ds-bio DNA/well; [2] 500 ng/ml ds-bio DNA/well	annealing of 30 bp long DNA - 10 bp 5' end overhang; filling overhang with Klenow Fragment and dNTPs with 11-biotin-UTP; 7-8 biotin molecules per ds-DNA; 0.04-0.4 pmol ds-bio DNA/reaction	annealing of 27 bp long DNA results in sticky ends - ligation; biotinylation; 10 µg ds-bio DNA/well
**DNA-ELISA Procedure**	Streptavidin-coated microplates 96 well (Thermo Scientific) - DNA immobilisation	StreptaWell transparent (Boehringer) coated with 5µg/ml Streptavidin (250 ng/well) - immobilisation of antibody bound p53 protein incubated with ds-bio DNA	Streptavidin coated plates (Roche) - immobilisation protein	high-binding 96-well plates [1] (Costar) coated with GST:6D3 in PBS - immobilisation of protein or [2] Reacti-Bind streptavidin-coated plates (Pierce Chemical) - immobilisation of DNA	Immunolon 2 plate (Dynatech) coated with ant mouse or anti-rat IgG - immobilisation of antibody bound p53 protein incubated with ds-bio DNA	MaxiSorb Immuno plates (NUNC) coated with Streptavidin - DNA immobilisation
Protein Binding Buffer	extraction buffer or TBS-T (protein dependent)	5 mM Tris, 0.5 mM EDTA, 50 mM KCl (pH 7.8)	4 mM HEPES pH 7.5, 100 mM KCl, 8% glycerol, 5 mM DTT, 0.2% BSA, 0.016% poly d(IC)	PBS	100mM NaCl, 20 mM Tris-HCl, pH7.5, 10% glycerol, 1% NP40, 5mM DTT, 0.1-1µg poly(dI:dC)	protein extraction buffer
Wash Steps	TBS-T or PBS-T	PBS + 0.1% Tween20 (140 mM NaCl, 3 mM KCl, 4 mM Na_2PO_4)	PBS + 0.1% Tween20	-	PBS; 0.1% NP40 in 20mM Tris-HCl pH 7.5	PBS-T
Blocking Solution	5% non-fat dried milk (Roth) in TBS-T or antibody related blocking solution (Qiagen) (antibody dependent)	3% BSA in PBS	no extra step	5% skim milk (Difco) [1]	20% non-fat dry milk, 1mg/ml heat-denat salmon sperm DNA	-
Antibodies for Detection of Protein-DNA-Interaction	anti-His conjugated with horseraddish peroxidase (Qiagen)	primary mouse anti-p53 antibody in protein-binding buffer, secondary with horseraddish peroxidase	primary rabbit anti-NFkB antibody (1:1000 in 10 mM pBS pH 7.4, 50 mM NaCl, 1% non-fat dried milk), secondary with horseraddish peroxidase	streptavidin- and biotin-substituted horseradish peroxidase or primary against GST, secondary with horseradish peroxidase	streptavidin conjugated alcaline phosphatase	primary against E2, seconday with peroxidase (Dakopatts)
Signal Detection	4 mg OPD (Sigma), 0.001% (v/v) H_2O_2 in 6 ml CP-buffer (10 mM Na_2HPO_4, 100 mM citric acid, pH5); after 20 min stop with 1:1 2 M HCl; 492 nm with 650 nm reference	ABTS (Chemicon International), 405 nm	Tetramethylbenzidine (Biosource), 450 nm with 655 nm reference, avoiding of saturation 405 nm	ABC detection kit (Dako), 492 nm [1]	1mg/ml p-nitrophenyl phosphate in 10mM diethanolamine pH 9.8, 0.5mM $MgCl_2$; 405 nm	4 mg OPD, 0.03% H_2O_2 in 10 ml 0.2M phosphate-citrate buffer pH5; after 20 min stop with 1:1 H_2SO_4; 490 nm
**Results**	applicable for any (plant) transcription factor o DNA-binding protein/domain	linker of 5 bp before and after 20 bp long core DNA sequence better than only 20 bp sequence or 58 bp long promoter sequence	DNA-ELISA 10-fold more sensitive than EMSA, competion of protein-DNA interaction with non-bio-ds DNA possible	DNA-ELISA 10-fold more sensitive than EMSA; ELISA-procedure [1] is more sensitive than [2], because of sterical reasons	specific quantitation of functional protein in a sample	applicable for any DNA binding protein, assay is oligonucleotide and protein specific
